# Experimental models for the study of neurodegeneration in amyotrophic lateral sclerosis

**DOI:** 10.1186/1750-1326-4-31

**Published:** 2009-07-20

**Authors:** Luis B Tovar-y-Romo, Luz Diana Santa-Cruz, Ricardo Tapia

**Affiliations:** 1 División de Neurociencias, Instituto de Fisiología Celular, Universidad Nacional Autónoma de México, AP 70-253, 04510-México, D.F., México

## Abstract

Amyotrophic lateral sclerosis (ALS) is a fatal neurodegenerative disease of unknown cause, characterized by the selective and progressive death of both upper and lower motoneurons, leading to a progressive paralysis. Experimental animal models of the disease may provide knowledge of the pathophysiological mechanisms and allow the design and testing of therapeutic strategies, provided that they mimic as close as possible the symptoms and temporal progression of the human disease. The principal hypotheses proposed to explain the mechanisms of motoneuron degeneration have been studied mostly in models in vitro, such as primary cultures of fetal motoneurons, organotypic cultures of spinal cord sections from postnatal rodents and the motoneuron-like hybridoma cell line NSC-34. However, these models are flawed in the sense that they do not allow a direct correlation between motoneuron death and its physical consequences like paralysis. In vivo, the most widely used model is the transgenic mouse that bears a human mutant superoxide dismutase 1, the only known cause of ALS. The major disadvantage of this model is that it represents about 2%–3% of human ALS. In addition, there is a growing concern on the accuracy of these transgenic models and the extrapolations of the findings made in these animals to the clinics. Models of spontaneous motoneuron disease, like the wobbler and pmn mice, have been used aiming to understand the basic cellular mechanisms of motoneuron diseases, but these abnormalities are probably different from those occurring in ALS. Therefore, the design and testing of in vivo models of sporadic ALS, which accounts for >90% of the disease, is necessary. The main models of this type are based on the excitotoxic death of spinal motoneurons and might be useful even when there is no definitive demonstration that excitotoxicity is a cause of human ALS. Despite their difficulties, these models offer the best possibility to establish valid correlations between cellular alterations and motor behavior, although improvements are still necessary in order to produce a reliable and integrative model that accurately reproduces the cellular mechanisms of motoneuron degeneration in ALS.

## Introduction

Effective treatments for practically all diseases can only result from the knowledge of their cellular and molecular pathophysiological mechanisms. This is particularly evident in the case of diseases whose cause is still unknown in spite of the remarkable progress of biomedicine in the recent decades, such as devastating neurodegenerative diseases, including Alzheimer's disease and amyotrophic lateral sclerosis (ALS). For the purpose of gaining insights into such mechanisms, the design and use of experimental models is essential. In general, such studies are carried out in vitro, in cell cultures, slices or organotypic cultures, and in vivo. Whereas the former can give very useful information regarding cellular and molecular mechanisms, the experiments in whole living animal models obviously reflect more closely the human disease, provided that the symptoms and their development during time mimics as close as possible those of the human disease. In this framework, the purpose of the present article is to review the available experimental animal models of ALS.

Amyotrophic lateral sclerosis, described in 1869 by the French neurologist Jean-Martin Charcot, is a fatal adult-onset neurodegenerative disease characterized by the selective and progressive death of both upper and lower motoneurons, leading to a progressive paralysis, respiratory depression and death usually within 2–5 years after onset. Based on which type of motoneurons are primarily affected, whether lower motoneurons, located in the ventral horns of the spinal cord, or upper motoneurons, located in the brainstem and the cerebral motor cortex, ALS can be classified in two forms: spinal onset (~75% of cases), characterized by muscle weakness and atrophy, cramps, fasciculations, spasticity and paralysis, and bulbar-onset (~25% of cases), characterized by progressive dysphagia and dysarthria, spasticity and hyperreflexia [1]. Because the neuronal loss in ALS is selective, the disease generally does not cause major cognitive impairments such as those occurring in other neurodegenerative diseases like Alzheimer's and Huntington's. However, some ALS patients may present changes in personality, irritability, obsessions, poor insight and deficits in frontal executive tests [2]. In the majority of ALS patients, death is due to respiratory failure caused by the denervation of the respiratory muscles and diaphragm. The prevalence of ALS is about 2–6 cases/100,000 and the median age of onset is 55 years, although it can start at younger ages [3].

The disease occurs in sporadic and familial forms with very similar clinical courses and common pathological features, such as the presence of abnormal accumulations of neurofilaments in degenerating motoneurons [4]. The familial form of ALS (FALS) accounts for 5–10% of cases and has an autosomal dominant pattern of inheritance, whereas the sporadic form (SALS) accounts for the majority of ALS cases (~90%). Among the FALS cases, about 20% are caused by missense mutations in the *SOD1 *gene that codes for the enzyme Cu^2+^Zn^2+ ^superoxide dismutase 1 (SOD1) [5]. However, the cause of most ALS cases is still unknown and several hypotheses have been proposed to account for the selective death of upper and lower motoneurons. These include oxidative damage, axonal strangulation and transport impairment, disorganization of neurofilaments, protein misfolding and toxicity from intracellular aggregates, mitochondrial dysfunction, inflammation, apoptosis, and excitotoxic death arising from the mishandling of glutamate [4,6-10]. Because the clinical course of the disease is highly variable, the mechanism of motoneuron death may arise from the unfortunate convergence of multiple factors rather than from a single alternative.

The pathogenesis of ALS has been studied in autopsy samples, but this has not yielded reliable information in terms of the pathophysiological mechanisms of motoneuron degeneration during the progressive clinical stage, from disease onset to the death of the patients. The pathological hallmarks that have been found in the spinal cord of autopsied ALS patients include the atrophy of dying motoneurons with notable swelling of the perikarya and proximal axons, intracytoplasmic neurofilament abnormalities, and the presence of Bunina bodies, spheroids and strands of ubiquitinated material in degenerating axons and in cell somas; this motoneuron pathology is often accompanied by reactive gliosis [11].

Under these circumstances, experimental in vitro and in vivo models have been developed to improve our understanding of the disease and have allowed the testing of possible therapeutic strategies. However, these models have many limitations and have not succeeded in designing effective treatments to stop the course of the disease. Therefore, an integrative model that reproduces the chronic progressive motoneuron death and the main characteristics of the disease is still needed.

Based on the multiple events considered to contribute to the selective loss of motoneurons as targets for therapy, many different drugs have been tested on their capacity to alleviate or retard the symptoms of ALS patients and to prolong their survival, but none has proved to be effective. The only currently used compound that slightly slows disease progression and prolongs the survival of ALS patients, with no improvement in muscle function, is riluzole. This drug limits glutamate release from nerve endings possibly by stabilizing the inactive state of voltage-dependent sodium channels and by a G protein-coupled intracellular pathway [12-16].

## Mutations in superoxide dismutase 1 as cause of one form of familial ALS

Cu^2+^/Zn^2+ ^superoxide dismutase 1 (SOD1) is an ubiquitously expressed cytoplasmic enzyme that catalyzes the dismutation of the superoxide radical (O_2 _^-^) into hydrogen peroxide and molecular oxygen and is an important free radical scavenging enzyme that protects cells against oxidative stress. The copper atom is alternately reduced and oxidized by superoxide, providing a reactive center for its dismutation, while the zinc atom gives structural stability to the protein; both cations are buried at the bottom of an active site channel [17-19]. The disease-causing SOD1 mutations are scattered throughout the primary structure of the protein. More than 100 mutations have been found [20-22], and all but one, SOD1^D90A ^[23,24], cause the dominantly inherited disease. Superoxide radical is a very reactive intermediate formed by the reduction of O_2 _in the respiratory chain and is a powerful oxidant; it is normally converted to hydrogen peroxide before it can undergo other free-radical reactions. Hydrogen peroxide is converted to water by catalase or glutathione peroxidase, but it can also be decomposed to hydroxyl radical in the presence of iron; this radical is highly reactive and can damage lipids, proteins or nucleic acids.

It has been demonstrated that SOD1-mediated toxicity in ALS is not due to the loss of its catalytic activity but instead to a gain of function which confers one or more toxic properties that are independent of the levels of dismutase activity [25]. The main arguments against the importance of loss of dismutase function are that SOD1 knockout mice do not develop motoneuron disease [26] and that levels of SOD1 activity do not correlate with disease in mice or humans. In fact, some mutant enzymes retain full dismutase activity [27,28], and chronic increase in the levels of wild-type SOD1 (and dismutase activity) has no effect on the disease [29] or even accelerates it [30]. The acquired toxic property likely disrupts several basic cellular functions in neurons, including protein breakdown by the ubiquitin-proteasome system, slow anterograde transport, fast retrograde axonal transport, calcium homeostasis, mitochondrial function, and maintenance of the cytoskeletal architecture [7]. The toxicity can arise either through aberrant chemistry, mediated by the misfolded aggregated mutants, which can disregulate the redox equilibrium [31] or produce loss or sequestration of essential cellular components, for example by saturating the protein-folding chaperones and/or the protein-degradation machinery [7,8,31]. This includes endoplasmic reticulum stress and accumulation of the mutant SOD1 in microsomes [32,33]. The discovery of prominent cytoplasmic inclusions in motoneurons and, in some cases, within the astrocytes surrounding them in the SOD1 ALS mouse model [29,30,34] and in autopsy samples from patients with SALS and FALS [35-37] led to the hypothesis of toxic protein aggregation. These inclusions are commonly detergent-insoluble elements dispersed in the cytosol, which have been characterized by ubiquitin and SOD1 staining [38,39]. The toxicity to motoneurons generated by SOD1 mutants seems to be non-cell autonomous, since mutant damage occurs not just within motoneurons but also in non-neuronal cells, suggesting that neuronal death depends, at least in part, on a contribution from surrounding astrocytes and possibly other cell types [40-42].

## Other mutations as cause of familial ALS

A series of other mutant genes have been reported to cause ALS in both familial and sporadic cases (see [43,44] for comprehensive reviews), but the number of patients harboring these mutations is substantially low, and therefore these mutations are not commonly used for modeling ALS, although there are few exceptions such as alsin.

Alsin, the product of the *ALS2 *gene coded in chromosome 2q33 [45], is a protein with three putative guanine nucleotide exchange factor domains that has been found altered in some FALS cases [46,47], with a higher prevalence in Tunisian and Pakistani populations [45,48]. Most *ALS2 *mutations are deletions caused by abnormal stop codons that produce a truncated dysfunctional protein [49]. Homozygous expression of mutant alsin is responsible for early onset FALS, also known as juvenile ALS or ALS2; this form progresses slower than the adult onset forms [50]. Since juvenile ALS is inherited in a recessive manner it is assumed that the proper function of alsin is an elemental component of motoneuron physiology. Unlike SOD1 mutations that cause a rather homogeneous phenotype independently from the amino acid substitution, although subtle differences exist, *ALS2 *truncated products cause a diversity of clinical outcomes depending on the form generated by the specific mutation. Furthermore, mutations in alsin are not only responsible for provoking ALS but for at least two other types of motoneuron disease: primary lateral sclerosis and hereditary spastic paraplegia (reviewed in [51]).

Recently, it was reported that mutations in the RNA/DNA binding protein TDP-43 cause classical ALS with an autosomal recessive inheritance pattern (see [52] for review), and shortly after this discovery another nucleic acid binding protein was found to be mutated in a British family with FALS [53,54]. The discovery of these mutations may allow the development of new experimental models for ALS, although as we shall discuss later, such models might be limited to reproduce the causes of motoneuron death related to those specific mutations.

## Glutamate-mediated excitotoxicity as a causal factor of ALS

Glutamate-mediated excitotoxicity generated by an excessive glutamatergic synaptic transmission is considered a probable mechanism leading to motoneuron degeneration in both SALS and FALS. Excitotoxicity involves a massive influx of Ca^2+ ^through glutamate receptors, triggering the uncontrolled activation of deleterious processes that eventually produce neuronal death [55,56]. Motoneurons are highly vulnerable to intracellular calcium overload due to their low calcium buffering capacity [57-60].

In spinal motoneurons, calcium entry is likely to occur through calcium-permeable AMPA (α-amino-3-hydroxy-5-methylisoxazole-4-propionate)-type receptors. Functional AMPA receptors are composed of four subunits, GluR1-GluR4, in various combinations [61]. Subunit GluR2 determines the calcium permeability because the presence of at least one GluR2 in the receptor structure makes it impermeable to the cation [62,63]. In addition, the posttranscriptional modifications that edit the Q/R site of the GluR2 impede the calcium flux through the pore [64]. Therefore, activation of AMPA receptors lacking the GluR2 subunit or without its posttranscriptional edition may have significant pathophysiologic consequences that could be involved in ALS. In fact, spinal motoneurons are particularly vulnerable to agonists of the AMPA-type receptors probably because of a large calcium influx, both in vitro [56,65,67-72] and in vivo [73,74].

Glutamatergic synaptic transmission is rapidly terminated by the neurotransmitter uptake from the synaptic cleft into neurons and glia, a process carried out by high affinity glutamate transporters. Five of these transporters have been identified and cloned, and their location in the central nervous system has been determined. Excitatory amino acid transporter 2 (EAAT2), also called glutamate transporter 1 (GLT1) in rodents, is the most abundant and is present almost exclusively in astrocytes [75]. In ALS patients, post-mortem analysis of the motor cortex and the spinal cord revealed a reduction in the content of EAAT2 [69,76,77]. These findings led to the hypotheses that malfunction of EAAT2 might be an important cause of motoneuron death in ALS, but it is still unknown if the loss of glutamate transporters in the tissue of ALS patients is a cause or a consequence of neuronal loss [78]. In addition, even when increased levels of glutamate in the cerebrospinal fluid and plasma of SALS patients have been described [79,80], this increase occurs in only ~40% of patients [81,82], suggesting that elevated glutamate does not seem to be the triggering factor for motoneuron death in SALS. Furthermore, recent findings from our laboratory do not support this hypothesis, because the acute [73] and chronic [83] pharmacological blockade of glutamate transport in the rat spinal cord in vivo, which results in increased concentrations of extracellular glutamate, failed to cause motoneuron death or motor deficits. Also, no neuronal damage was observed in the hippocampus and motor cortex of transgenic FALS mice in which extracellular glutamate was elevated by transport blockade [84].

## Oxidative stress in ALS

There are many data supporting the involvement of oxidative stress in ALS pathogenesis. Analysis of post mortem tissue from ALS patients has revealed an increased oxidative damage of cell components as compared to controls, like oxidized DNA [85,86] and formation of carbonyl [85,87] and nitrotyrosine [88-90] derivatives in proteins. Furthermore, lipid peroxidation and protein glycoxidation were found increased in spinal cord motoneurons and glial cells [91]. Markers of oxidative damage have also been analyzed in cerebrospinal fluid and plasma from living ALS patients during the course of the disease, showing enhanced DNA oxidative damage [92,93], lipid peroxidation [94-97] and elevation of nitrotyrosine [98], although the latter result is controversial [99]. Increased oxidative damage in macromolecules has also been demonstrated in the transgenic mutant SOD1 mouse, [100-104] suggesting that oxidative stress could be involved in FALS pathogenesis. In models in vitro, spinal motoneurons exposed to an excitotoxic insult by stimulation of AMPA receptors produced a mitochondrial calcium overload that triggered mitochondrial depolarization and generation of ROS [105].

All these data support the hypothesis of oxidative stress as a mechanism that contributes to motoneuron injury in ALS, but it is still unclear whether oxidative stress is a cause or a consequence of the disease, since it may result from other cellular processes like excitotoxicity, mitochondrial dysfunction or protein aggregation.

## Protein aggregation in ALS

The aggregation of misfolded proteins leads to cellular degenerative processes that ultimately cause neuronal death. This kind of disturbances is well characterized for neurodegenerative diseases like Alzheimer's, Parkinson's and Huntington's diseases, whereas in ALS, besides the previously discussed aggregation of mutant SOD1 in FALS, some toxic intracellular inclusions have been described in both SALS and FALS. The best described are changes in neurofilament composition that generate alterations in perykaria and proximal axons of motoneurons, a pathogenic characteristic of ALS described several years ago [106]. In some cases mutations were found in the heavy subunit of neurofilaments [107-109], and transgenic mice harboring mutant or overexpressed neurofilament subunits H and L show a motoneuron pathology reminiscent of that occurring in ALS [110,111].

## Experimental models for the study of ALS

The rationale of the foregoing review on the advances in the knowledge of the mechanisms of motoneuron death and the hypotheses on the pathophysiology of ALS is to provide a framework for analyzing the experimental approaches that have been developed when attempting to create valid experimental models of the disease. These include experiments in vitro using diverse spinal cord preparations, and whole animal experiments, which include animals with spontaneous motoneuron degeneration, transgenic rodents, and animals in which spinal motoneuron death was produced with pharmacological tools.

### In vitro models

#### Spinal cord cultures

Primary spinal cord cultures have been established and used to study the morphological, biochemical and electrophysiological characteristics of motoneurons for many years [112]. Generally, the tissue for cellular culture is taken from 12–14 days-old rodent embryos or 6–7 days-old chicken embryos, the spinal cord is dissociated by mechanical and enzymatic procedures and then plated on matrix-coated dishes. Motoneurons are relatively easy to identify in culture due to their large size (25 to 30 μm of diameter), but because they are present in very small quantities in the spinal cord (in a transversal section at the lumbar segment of the rat spinal cord there are less than 25 motoneurons in each side), motoneuron enriched cultures are often used instead of mixed primary cultures. Motoneuron enrichment is achieved by several methods. For example, the cellular suspension obtained after spinal cord homogenization is centrifuged in a metrizamide cushion, that separates cell bodies by cellular densities; motoneurons have a relatively low cellular density and the enriched fraction is identified by biochemical analyses of acetylcholine production [113].

Further purification of the motoneuron population can be achieved by the immuno-recognition of the nerve growth factor receptor p75 that motoneurons express since early embryonic stages. A specific antibody for the p75 receptor is immobilized in a Petri dish on which a suspension of cells is poured, and motoneurons specifically adhere to the antibody coated surface increasing their concentration [114]. Because motoneurons require a large variety of trophic factors to survive, the enriched cultures are generally seeded on top of a spinal glial feeder layer, usually obtained from the same spinal tissues that motoneurons came from. Enriched motoneuron cultures can also be obtained by flow cytometry. Motoneurons are labeled with fluorescent tracers that are injected in the developing muscle and retrogradely transported by the axons to the neuronal somas in the spinal cord, and then the labeled cells can be sorted [115]. Another way to obtain these cultures is to express an enhanced green fluorescent protein under a specific motoneuron promoter in embryonic stem cells; marked cells can be sorted and then differentiated into motoneurons in culture [116].

Modeling a complex disease in such a reduced and limited system has a series of inconveniences, but still, important information on some intracellular mechanisms can be obtained by studying the physiology of motoneurons. For example, using these systems it was first demonstrated that motoneurons are particularly vulnerable to glutamatergic excitotoxicity trough AMPA receptors [66], that the toxicity underlying this process is mediated by Ca^2+ ^[71,105] and that glutamate preferentially stimulates the production of reactive oxygen species in motoneurons in comparison with other neuronal types of the spinal cord [117].

A major shortcoming of these systems is that the conditions in which motoneurons exist must be substantially modified. For instance, the mentioned particular vulnerability of motoneurons to AMPA in culture is only seen when extracellular calcium concentration is raised from physiological 2 mM to 10 mM [66,71,105]. Also, the fact that the ratio motoneurons:glia is altered is not trivial, since non-neuronal cell types play a fundamental role in the pathogenesis of this disease and accumulating experimental evidence indicates that ALS is a non cell-autonomous disease, meaning that its origin may be localized not only in motoneurons, but in the surrounding spinal cells as well [40,41,118-120]. Thus, although cultures of glial cells have helped to elucidate the participation of other cellular types in the development of ALS, like micro and astroglia toxicity due to the expression of mutant SOD1, the information obtained from these studies is limited to the particular experimental conditions employed.

#### NSC-34 cells

Establishing a cell line of immortalized neurons in culture is a difficult task, mainly because of the intrinsic properties of the neuronal lineage, including their null capacity to proliferate when they are fully differentiated. In an attempt to circumvent this problem, a hybrid cell line (NSC-34) of neuroblastoma (a highly proliferative neuronal cell type) and spinal cord motoneurons from enriched primary cultures, was produced by fusing the two cell types [121]. In this hybrid line some of the motoneuron characteristics are present, like acetylcholine synthesis, storage and release, action potential generation, expression of neurofilament proteins and association with neuromuscular synapse-specific basal lamina glycoproteins.

The NSC-34 line expressing mutant SOD1 is considered a cellular model of ALS. In these cells some features of motoneuron alterations have been described, for example, Golgi apparatus fragmentation [122], and mitochondrial dysregulation [123]. They have also been used to study in vitro some of the mechanisms of mutant SOD1 toxicity [124-126]. Nonetheless, NSC-34 cells also retain characteristics of the neuroblastoma linage, like an enhanced N-myc action [121]. N-myc is an oncogene that directs diverse cellular responses, especially those involved in proliferation [127]. For the study of the mechanisms of neuronal death and its prevention by possible therapeutic agents the effects of N-myc could obstruct the underling mechanisms, making this model faulty.

#### Organotypic cultures

All neurons exist in a specific tissue context where the surrounding cellular types shape the biochemical, electrophysiological and morphological characteristics of each neuron. Motoneuron excitatory or inhibitory inputs and outputs from their afferences and interneurons, as well as trophic support from surrounding glia and its reactivity in response to neuronal death, modulate important characteristics of the cellular and molecular processes that occur in ALS. When spinal cord is disgregated and plated on dishes, all these important cellular interactions are lost. One way to preserve the tissue structure for in vitro analyzes is to cultivate an entire slice of the spinal cord in an organotypic culture. With this method, spinal cord slices obtained from neonate pups are chopped into 400 μm thick sections that can be cultured for as long as 3 months. Motoneurons in this type of preparations retain their metabolic characteristics like choline acetyltransferase and acetylcholinesterase activities [128].

Slow motoneuron degeneration in organotypic cultures has been induced by the chronic exposure to the glutamate transporter blocker threo-β-hydroxyaspartate (THA) [129]. Using this system it has been reported that neurotrophic factors are able to protect motoneurons from excitotoxic death [130,131]. As with the cell cultures, a major shortcoming of organotypic systems is that they do not always accurately reproduce what would be happening in an in vivo system and even less in an ALS patient. For example, while the glutamate transport blockade is highly toxic for motoneurons in organotypic cultures [129-131], as already mentioned the blockade of glutamate uptake in vivo is innocuous for motoneurons [73,83,84].

### In vivo models

#### Spontaneous genetic defects that cause motoneuron degeneration

The oldest known model of spontaneous motoneuron alterations is the wobbler mouse. This mouse harbors a mutation in the *wr *locus that has been mapped in chromosome 11 [132]. It was recently found that this gene that codes for the vacuolar-vesicular protein sorting 54 (Vps54) has the missense mutation L967Q in the wobbler mouse [133]. Homozygous expression of the mutant *wr *gene causes the death of motoneurons in the cervical portion of the spinal cord, affecting mainly the somas, and in consequence, causing a proximal axonopathy [134]. The pathology presented by these animals may have a glial origin, since they develop astrocytic defects and an increased astrocyte reactivity that seems independent from motoneuron death [135].

The mechanisms of neurodegeneration in the wobbler mice are so far unidentified. Excitotoxic processes seem not be involved, since glutamate transporters are expressed normally, extracellular glutamate levels are unchanged [136], and AMPA receptor antagonists have no effect on motor behavior and neuronal loss [137]. Also, results of TUNEL staining of fragmented DNA [138], and activation of caspases suggest that apoptosis is not involved in neuronal death in these mice [139].

Another model of spontaneous motoneuron death is a mouse that suffers a progressive motoneuronopathy (pmn), caused by the homozygous expression of the mutant tubulin-specific chaperone E [140,141]. These animals develop a progressive caudo-cranial degeneration of the motor axons and die at few weeks after birth [142]. The main pathological characteristic of the pmn mouse is a distal axonopathy with minor alterations of neuronal somas, an alteration different from the ALS pathology [143]. Although the pathophysiology of motoneuron death in these animals is not the same as in ALS, the neuroprotective potential of some agents, like certain trophic factors [144,145] or anti-excitotoxic compounds [146] have been shown to be partially effective.

The wasted mutant mouse with genotype *wst*/*wst *is another murine model of spontaneous spinal neurodegeneration. These animals develop a hindlimb paralysis, not necessarily due to motoneuron loss but to cell vacuolization [147]. There are reported cases of spontaneous motoneuron disease in larger animals. A spontaneous disease of the horse, known as equine motoneuron disease, is characterized by a generalized weakness, progressive muscle atrophy and loss of motoneurons in the spinal cord and brainstem [148]. In dogs, a condition named hereditary canine muscular atrophy is caused by a mild loss of motoneurons in spinal cord and brainstem and neurofibrillary swellings in proximal axons [149]. These animals may mimic in some way the pathology underlying sporadic ALS, but their use in experimental studies is clearly complicated.

#### Genetically modified models

##### - Alsin knock out mice

Human mutations in the alsin protein encoded by the *ALS2 *gene in some FALS cases were mentioned in a previous section. *ALS2 *knock out mice have been developed and shown to be deficient in motor coordination and motor learning [150], to have abnormalities in endosome trafficking [151,152], as well as axonal degeneration [153-155] and increased susceptibility to oxidative stress [150]. Although no overt motoneuron degeneration is present, these animals appear to be a good in vivo model for the study of the alterations of motoneuron physiology that take place before cellular death such as axonal impairment, but, as in the case of mutant SOD1 transgenic mice described next, the processes studied may mimic only those occurring in patients with this type of genetic alterations.

##### - Transgenic mutant SOD1 rodents

A major breakthrough in the ALS research was the discovery that ~20% FALS cases were due to mutant SOD1 [5]. Almost immediately after this finding, transgenic mice that express mutant human SOD1 were developed. The initial mutations produced the substitution of glycine for alanine at position 93 (G93A), and of alanine for valine at position 4 (A4V) [156]. Other SOD1 mutations have been expressed in transgenic mice. The commonest, in addition to G93A and A4V, are glutamate for arginine substitution at positions 37 (G37R) [157] and 85 (G85R) [158]. The phenotype expressed by these mice, regarding the age at symptoms onset and their severity is directly proportional to the amount of mutant protein expressed in the tissue [159], lending further support to the hypothesis of the acquired toxic function of mutant SOD1 mentioned before.

The disease in transgenic mice begins with hindlimb weakness, impaired leg extension and shortened stride length, and continues to complete paralysis of the limbs, principally the rear ones, within few days [156]. Cellular alterations are mainly characterized by vacuolar degeneration of motoneurons and their processes at the early stages, followed by neuronal loss and atrophy of the ventral horns in the spinal cord at the late stages; the most damaged tissue is the spinal cord, but the medulla, pons and midbrain are affected as well [34]. In addition, mice expressing the G85R mutation in the murine SOD1 develop paralysis due to motoneuron loss similar to that presented by the transgenic expression of human mutant SOD1 [160]. Transgenic rats expressing mutant human SOD1 have also been developed and display a similar pathology [161,162].

Since no symptomatic or pathological evident differences exist between FALS and SALS, it has been assumed that the mechanisms underlying both types of the disease are shared. Therefore, transgenic SOD1 animals have been extensively used in numerous studies related to ALS, principally because the phenotype they display is elicited by the only proven cause of the disease. In this FALS model all the primary hypothesis on ALS origin have been put to challenge, including oxidative stress [31,163,164], excitotoxicity [84,165], apoptosis [39,166,167], protein aggregation [39], axonal dysfunction [168], mitochondrial failure [40,166,167], endoplasmic reticulum stress [32,33,169], and practically every other suspected mechanism. This model has also been the golden standard for drugs and therapies testing at experimental stages. Nonetheless, the major weakness of this FALS model is that it reproduces the mechanism of neuronal death that occurs in only ~2% of ALS cases. Therefore, it is not completely accurate to extrapolate the findings made in this model to the processes occurring in patients that do not bear mutations in SOD1 [165]. In addition, a meta-analysis of a vast number of data on the therapeutic action of many drugs in SOD1 mutant mice revealed that most of these experiments show serious methodological problems, such as a bias for reporting beneficial effects, small number of animals, lack of randomization and blinding and the initiation of the treatment prior to symptoms onset, something that cannot be done in human ALS [170]. These problems may be the reason for the lack of reproducibility of the therapeutic effect of several drugs when tested in large cohorts and in blind manner, as was described in detail in another recent study [171]. This clearly urges the necessity to test these potential treatments in non familial related models of motoneuron degeneration.

#### Models of non-genetic spinal motoneuron degeneration

There are much less animal models for SALS than for FALS, in spite of the fact that it accounts for >90% of the cases. Taking advantage of the knowledge on motoneuron death by excitotoxicity discussed above, our group has developed some acute and chronic experimental models that should be useful for studying the mechanisms of spinal motoneuron death and for testing potential therapeutic agents. The objective of the first experiments in this effort was to test whether the excitotoxicity produced by N-methyl-D-aspartate and AMPA/kainate receptor agonists or by endogenous glutamate might induce spinal motoneuron death and paralysis in the living rat. The experimental procedure used was microdialysis in the lumbar spinal cord, which permits the perfusion of drugs and at the same time the collection of extracellular fluid for the measurement of glutamate and other amino acids. Using this method we found that increasing the concentration of endogenous extracellular glutamate through the inhibition of glutamate transport failed to cause motor alterations or motoneurons loss, results that do not support the hypothesis of glutamate-mediated excitotoxicity resulting from the loss of glutamate transporters. On the other hand, perfusion of AMPA produced permanent paralysis of the ipsilateral hindlimb and a remarkable loss of spinal motoneurons, which started ~3–6 h after the beginning of the perfusion and progressed until reaching a maximum at 12 h, when practically all motoneurons in the spinal segment studied were lost. All these effects were prevented by the AMPA receptor antagonist 2,3-dihydroxy-6-nitro-7-sulfamoyl-benzo(F)quinoxaline (NBQX) and by an inhibitor of proteases [73,172].

The fact that the motoneuron death in our model begins only 3–6 h after the infusion of AMPA, and becomes complete at 12–24 h led to the hypothesis that the entry of calcium, probably through the calcium-permeable AMPA receptor channel, induces a delayed deleterious process leading to motoneuron death. To test this hypothesis, AMPA was co-applied with 1-naphthyl acetyl spermine (NAS) a selective blocker of the AMPA receptor that lacks the GluR2 subunit [67,173-175]. This compound significantly prevented the selective spinal motoneuron loss and the subsequent paralysis [74], indicating that rat spinal cord motoneurons possess functional calcium-permeable AMPA receptors lacking GluR2 and suggesting that the cellular process leading to motoneuron death in this model, in vivo, is triggered by an increase of intracellular calcium via these receptors. The hypothesis that such an increase is responsible for the damage was confirmed by the co-perfusion of the intracellular calcium chelator 1,2-bis-(o-aminophenoxy)-ethane-N,N,-N',N'-tetraacetic acid tetraacetoxy-methyl ester (BAPTA-AM) with AMPA, which was as effective as NAS in the prevention of the motoneuron damage and the paralysis [74]. The relevance of these findings is noteworthy because, differently from the experiments in vitro mentioned in a previous section, the neuronal death due to increased cytoplasmic Ca^2+ ^occurs under the physiological extracellular concentration of the cation (2 mM). Altogether, these data suggest that AMPA receptors may have an important role in the development of ALS.

The main limitation of the model discussed above is that the paralysis develops within a few hours, whereas motoneuron death in ALS is a chronic process that takes lengthened time periods. For this reason, we designed a different experimental approach, allowing the continuous slow infusion of AMPA in the spinal cord during several days using osmotic minipumps. This procedure generated a chronic model of spinal motoneuron degeneration induced by excitotoxicity, because it produced progressive motor impairment and motoneuron death along several days, depending on the AMPA concentration, resembling the characteristics of neurodegeneration and paralysis that are present in both ALS patients and FALS rodents [176]. Interestingly, we demonstrated that the coinfusion of vascular endothelial growth factor (VEGF) with AMPA remarkably protected against the deleterious chronic effect of the latter, indicating that this chronic model may indeed be useful for testing therapeutic strategies for ALS [176].

We have thus developed in vivo models of acute and chronic spinal motoneuron degeneration, in the absence of altered genetic components, which are useful for studying the mechanisms of this degeneration and for assaying potential neuroprotective compounds. As discussed throughout this article, this is relevant in view of the need of experimental animal models that reproduce motoneuron degeneration in processes such as sporadic ALS, which are not related to genetic alterations and occur in the great majority of ALS cases. The main limitation of our model is that, although glutamate-mediated overactivation of AMPA receptors may be a relevant mechanism of spinal motoneuron degeneration in ALS, convincing evidence that this occurs in the human disease has not yet been obtained.

## Conclusion

In spite of the high complexity of ALS, it is clear that great progress has been made regarding the mechanisms of motoneuron death. However, one of the strongest obstacles is the insufficient information on the cause of the selectivity of motoneuron degeneration, because many of the postulated mechanisms are common for the death of other types of neurons located in several brain regions. This is the case, for example, of Parkinson's and Alzheimer's diseases. The intracellular Ca^2+^-dependent neuronal death via overactivation of Ca^2+^-permeable AMPA receptors may be an important factor for this selectivity, because of the abundance of this type of glutamate receptors in spinal cord motoneurons.

It is also evident that valuable data have been obtained from experimental approaches in vitro, and that the combination of these results with those generated in models in vivo may lead to a better understanding of the pathophysiology of the disease and therefore to the design of effective therapeutic measures. Nevertheless, it appears that the limitations of the models in vitro are far greater than those of in vivo models, despite the difficulties of the experiments in vivo and their interpretation, correlations between cellular alterations and motor behavior can only be obtained in the whole animal.

Most of the experimental models of ALS are in fact FALS models, because they are transgenic rodents expressing human mutant SOD1. It is therefore very relevant to develop in vivo models of SALS, responsible for >90% of all ALS cases. Such models can be generated by applying the limited knowledge on the mechanisms already available, such as those related to excitotoxicity.

## Competing interests

The authors declare that they have no competing interests.

## Authors' contributions

LBTR, LDSC and RT reviewed the literature, wrote, read and approved the final manuscript.
